# 超高效液相色谱-电雾式检测器测定福建产绞股蓝中绞股蓝皂苷XLVI和LVI

**DOI:** 10.3724/SP.J.1123.2022.01024

**Published:** 2022-09-08

**Authors:** Pengxin LU, Gang LI, Wei ZHENG, Haizhen LIANG, Jie ZHANG, Ruiping CHAI, Dingqiang LUO, Yan JIN, Baolin GUO, Baiping MA

**Affiliations:** 1.广东药科大学中药学院, 广东 广州 510060; 1. School of Traditional Chinese Medicine, Guangdong Pharmaceutical University, Guangzhou 510060, China; 2.军事科学院军事医学研究院辐射医学研究所, 北京 100850; 2. Institute of Radiation Medicine, Academy of Military Medical Sciences, Academy of Military Sciences, Beijing 100850, China; 3.赛默飞世尔科技(中国)有限公司, 上海 201206; 3. Thermo Fisher Scientific (China) Co., Ltd., Shanghai 201206, China; 4.中国医学科学院北京协和医学院药用植物研究所, 北京 100193; 4. Institute of Medicinal Plant Development, Chinese Academy of Medical Sciences and Peking Union Medical College, Beijing 100193, China; 5.陕西省食品药品检验研究院, 陕西 西安 710065; 5. Shaanxi Institute for Food and Drug Control, Xi'an 710065, China

**Keywords:** 超高效液相色谱-电雾式检测器, 绞股蓝皂苷XLVI, 绞股蓝皂苷LVI, 福建产绞股蓝, ultra-high performance liquid chromatography-charged aerosol detector (UHPLC-CAD), gypenoside XLVI, gypenoside LVI, *Gynostemma pentaphyllum* (Thunb.) Makino from Fujian

## Abstract

该研究利用超高效液相色谱-电雾式检测器(UHPLC-CAD)建立了福建产绞股蓝中绞股蓝皂苷XLVI和LVI含量的测定方法。首先利用超高效液相色谱-四极杆-飞行时间质谱(UHPLC-Q-TOF/MS)结合UHPLC-CAD鉴定了福建产绞股蓝的主要成分,其中绞股蓝皂苷XLVI、LVI以及二者相应的含丙二酰基酸性皂苷为其主成分,因此在含量测定时先进行碱水解预处理将酸性皂苷转化为对应的去丙二酰基中性皂苷,再利用UHPLC-CAD测定碱水解后绞股蓝皂苷XLVI和LVI的含量。将绞股蓝样品粉末在乙醇-水-氨水(50∶46∶4, v/v/v)和料液比1∶150(g∶mL)条件下超声提取30 min,静置24 h后,在Waters ACQUITY UPLC BEH C18色谱柱(100 mm×2.1 mm, 1.7 μm)上分离,采用0.1%(v/v)甲酸水溶液和乙腈作为流动相进行梯度洗脱,流速0.5 mL/min,柱温40 ℃,电雾式检测器检测。结果表明,绞股蓝皂苷XLVI和LVI分别在9.94~318.00 μg/mL和12.78~409.00 μg/mL范围内具有良好的线性关系,相关系数(*r*)分别为0.9993和0.9995。方法精密度、重复性和24 h稳定性试验的相对标准偏差(RSD)均小于2.0%(*n*=6),绞股蓝皂苷XLVI与LVI的加标回收率分别在100.2%~107.2%与97.9%~104.2%范围内,RSD值分别为2.4%与2.6%。16批绞股蓝样品含量测定结果显示:绞股蓝皂苷XLVI含量占0.57%~2.57%,绞股蓝皂苷LVI含量占0.66%~2.99%。该方法灵敏度高,重复性好,可用于福建产绞股蓝的质量研究和质量控制。

## Abstract (en)

*Gynostemma pentaphyllum* (Thunb.) Makino contains dammarane-type triterpenoid saponins, similar to ginseng, with a host of pharmacological activities. However, its planting resources and chemical composition are quite complex. The chemical constituents of *Gynostemma pentaphyllum* vary drastically among different origins and varieties. Thus, the corresponding quality control methods also need to be different. Currently, limited information is available about the quality control of *Gynostemma pentaphyllum* from Fujian. A new method based on ultra-high performance liquid chromatography-charged aerosol detection (UHPLC-CAD) was established for the determination of gypenoside XLVI and LVI in *Gynostemma pentaphyllum*. The major components of *Gynostemma pentaphyllum* were characterized using UHPLC-quadrupole time-of-flight-mass spectrometry (UHPLC-Q-TOF/MS) combined with UHPLC-CAD. The results revealed gypenoside XLVI, LVI, and their corresponding malonyl-containing acidic saponins as the main components. However, malonylgypenoside XLVI and LVI can easily remove their malonyl group and convert to gypenoside XLVI and LVI during the application of *Gynostemma pentaphyllum*. In this study, the samples were pretreated using alkali hydrolysis to transform the acid saponins completely, and the final contents of gypenoside XLVI and LVI were determined via UHPLC-CAD.

The optimal alkaline hydrolysis, extraction, and liquid chromatography conditions were established. First, the alkaline hydrolysis conditions were optimized. The effects of the volume of ammonia and reaction time on the contents of gypenoside XLVI, LVI, malonylgypenoside XLVI, and LVI were examined. Malonylgypenoside XLVI and LVI could be transformed completely to gypenoside XLVI and LVI by standing for 24 h in an ethanol-water-ammonia (50∶46∶4, v/v/v) mixture. Furthermore, the extraction conditions were optimized. Next, effects of the different solvents, extraction time, and solid-liquid ratio on the extraction rates of gypenoside XLVI and LVI were investigated. The extraction method for *Gynostemma pentaphyllum* powder using the ethanol-water-ammonia (50∶46∶4, v/v/v) and a solid-liquid ratio of 1∶150 (g∶mL) for 30 min was established. Finally, a prepared test solution was separated on a Waters ACQUITY UPLC BEH C18 chromatographic column (100 mm×2.1 mm, 1.7 μm). Acetonitrile and 0.1% (v/v) formic acid aqueous solution were used as the mobile phases for gradient elution. The flow rate was set to 0.5 mL/min and column temperature was maintained at 40 ℃. The separation was detected using a charged aerosol detector. Results indicated that the logarithm of the mass concentrations of gypenoside XLVI and LVI had a linear relationship with the logarithm of the peak area in the range of 9.94-318.00 μg/mL and 12.78-409.00 μg/mL, respectively. The correlation coefficients (*r*) were 0.9993 and 0.9995, respectively.

The limit of detection (LOD) and the limit of quantification (LOQ) of gypenoside XLVI were 1.58 μg/mL and 6.36 μg/mL, respectively. The LOD and LOQ of gypenoside LVI were 2.05 μg/mL and 8.18 μg/mL, respectively. The relative standard deviations (RSDs) of precision, repeatability, and 24 h stability were less than 2.0% (*n*=6). The spiked recoveries of gypenoside XLVI were 100.2%-107.2% and the RSD value was 2.4%. The spiked recoveries of gypenoside LVI were 97.9%-104.2% and the RSD value was 2.6%. The results of 16 batches of *Gynostemma pentaphyllum* samples indicated that the gypenoside XLVI content was 0.57%-2.57%, and gypenoside LVI content was 0.66%-2.99%. Hence, this method has high sensitivity and good reproducibility. Therefore, it can be used for quality research and quality control of *Gynostemma pentaphyllum* from Fujian.

绞股蓝(*Gynostemma pentaphyllum*(Thunb.) Makino)为葫芦科绞股蓝属多年生草质藤本植物^[1]^,最早作为民间食用野菜,见于《救荒本草》,后收录于《本草纲目》。现代药理学研究表明,绞股蓝具有降血脂^[2]^、降血糖^[3,4]^、抗缺氧^[5]^、保护神经^[6,7]^和抗肿瘤^[8]^等药理活性。植物化学研究发现其主要成分为与人参相似的达玛烷型三萜皂苷,因而被誉为“南方人参”,目前已从绞股蓝中分离得到328个皂苷类成分^[9]^,其中包括人参皂苷Rb_1_、Rb_3_、Rd和F_2_^[10]^。在我国,绞股蓝主要分布于秦岭和长江以南的广大地区,其中陕西、福建、广西和湖南等地为其主产区。国内药物原料绞股蓝总皂苷所使用的药材多来源于秦巴山区,张蒙蒙等^[11]^利用超高效液相色谱-四极杆-飞行时间质谱(UHPLC-Q-TOF/MS)鉴定了秦巴山区产绞股蓝主要的皂苷成分,发现其皂苷类成分具有以下结构特征:C-2位和C-12位不连有羟基;糖基连接在C-3位或C-21位,且由木糖基、阿拉伯糖基、鼠李糖基和葡萄糖基组成;C-17位侧链多成环。而福建产区的种植绞股蓝大多为日本引进品种201(甜味),总皂苷含量高^[12]^,同时由于其口味佳,目前是绞股蓝商品茶的主要来源。根据福建产绞股蓝的植物化学研究^[13⇓-15]^,其主要皂苷成分的结构特征为:C-2位和C-12位连有羟基;糖基连接在C-3位或C-20位,且由木糖基和葡萄糖基组成;C-17位侧链为直链。福建产绞股蓝与秦巴山区以及其他产区的绞股蓝的皂苷成分之间存在显著差异,这也被翟新房等^[16]^的研究所证实,相应的质量控制方法应有所不同。

绞股蓝尚未收录至2020版《中国药典》,仅收录于地方标准,如在《福建省中药材标准(2006年)》中,对绞股蓝的质量控制仅局限于性状、色味,对绞股蓝总皂苷或具体皂苷无明确的含量控制。根据文献调研发现^[17⇓⇓-20]^,目前关于绞股蓝质量控制的液相色谱法,多采用紫外(ultraviolet, UV)检测器测定绞股蓝中一种或多种皂苷的含量。然而皂苷类化合物通常无紫外吸收或仅为末端吸收,UV对于这类化合物的检测不稳定,灵敏度低,并且在梯度洗脱时容易出现基线漂移。电雾式检测器(charged aerosol detector, CAD)是一种质量相关的通用型检测器^[21]^,对比其他通用型检测器如蒸发光散射检测器(evaporative light scattering detector, ELSD)和示差折光检测器(differential refractive index detector, RID), CAD具有灵敏度更高^[22]^、重现性更好和检测范围更宽^[23]^等特点,其检测信号不依赖于被测物质的化学结构,更适用于无紫外吸收或只有较弱紫外吸收成分的定量分析^[24]^。

本研究利用UHPLC-Q-TOF/MS结合UHPLC-CAD鉴定了福建产绞股蓝的主要成分,经过碱水解后^[25]^,绞股蓝皂苷XLVI(gypenoside XLVI)和绞股蓝皂苷LVI(gypenoside LVI)含量远高于其他皂苷成分,为福建产绞股蓝中的主成分,适合作为福建产绞股蓝质量评价的指标成分,且目前缺少相关报道。因此,本研究利用UHPLC-CAD建立了福建产绞股蓝中绞股蓝皂苷XLVI和LVI的含量测定方法,为福建产绞股蓝药材的质量评价和标准制定提供理论参考。

## 1 实验部分

### 1.1 仪器、试剂与材料

Vanquish Flex UHPLC液相色谱仪、CAD检测器(美国Thermo Fisher公司); Waters ACQUITY I-Class超高效液相色谱系统、VION-IMS-Q-TOF质谱系统(美国Waters公司); BP 211D十万分之一天平(德国Sartorius公司); KQ-600DE数控超声波清洗器(昆山市超声仪器有限公司)。

乙腈(质谱纯,美国Thermo Fisher公司);蒸馏水(广州屈臣氏有限公司);无水乙醇(分析纯,国药集团化学试剂有限公司)。对照品绞股蓝皂苷XLVI和LVI由本课题组从福建产绞股蓝中分离得到,经HR-ESI-MS以及^1^H-NMR、^13^C-NMR等波谱分析鉴定结构,经UHPLC-CAD面积归一化法测得纯度均大于98.0%,结构式如图1所示。

**图 1:**
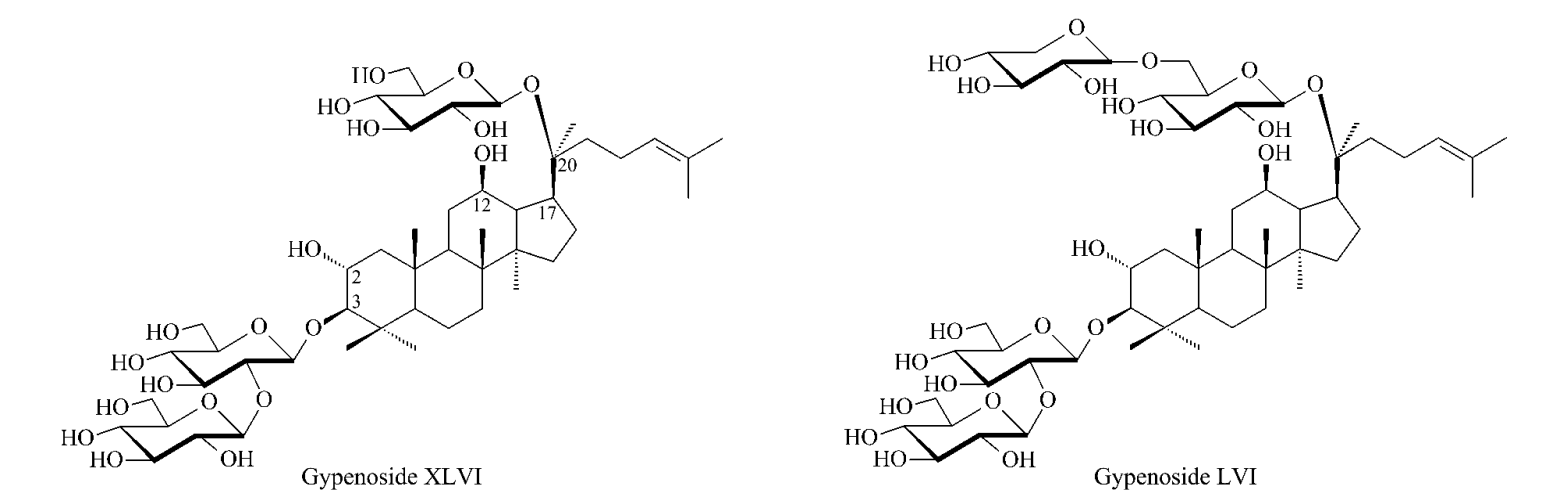
绞股蓝皂苷XLVI和LVI的结构式

药材来源信息见表1, 16份样品均来自福建绞股蓝主产区,经中国医学科学院药用植物研究所郭宝林研究员鉴定为葫芦科绞股蓝属植物绞股蓝。所有药材经烘箱50 ℃干燥至恒重后,粉碎,过40目筛得样品粉末,干燥阴凉处储存备用。

**表 1:** 绞股蓝样品信息

No.	Source	Specification	Wild or cultivation	Collection time
S1	Nanjing, Fujian (福建南靖)	crude drug	wild	2018.09
S2	Nanjing, Fujian (福建南靖)	crude drug	wild	2018.12
S3	Nanjing, Fujian (福建南靖)	crude drug	cultivation	2019.10
S4	Nanjing, Fujian (福建南靖)	crude drug	cultivation	2019.10
S5	Nanjing, Fujian (福建南靖)	crude drug	cultivation	2019.10
S6	Nanjing, Fujian (福建南靖)	crude drug	wild	2019.10
S7	Nanjing, Fujian (福建南靖)	crude drug	cultivation	2019.10
S8	Nanjing, Fujian (福建南靖)	crude drug	cultivation	2019.10
S9	Nanjing, Fujian (福建南靖)	crude drug	cultivation	2019.10
S10	Nanjing, Fujian (福建南靖)	crude drug	wild	2019.10
S11	Nanjing, Fujian (福建南靖)	crude drug	cultivation	2021.05
S12	Nanjing, Fujian (福建南靖)	crude drug	cultivation	2021.05
S13	Nanjing, Fujian (福建南靖)	crude drug	cultivation	2020.08
S14	Changtai, Fujian (福建长泰)	commercial tea	cultivation	2021.06
S15	Changtai, Fujian (福建长泰)	commercial tea	cultivation	2021.09
S16	Changtai, Fujian (福建长泰)	commercial tea	cultivation	2021.06

### 1.2 定性分析

#### 1.2.1 定性分析样品的配制

称取绞股蓝皂苷XLVI和LVI对照品粉末适量,分别溶解于乙醇-水(70∶30, v/v)中,各吸取一定体积均匀混合,0.22 μm微孔滤膜过滤即得对照品溶液。

精密称取绞股蓝样品粉末0.2 g,加入乙醇-水(70∶30, v/v)30 mL于具塞锥形瓶中,超声提取30 min,取上清液经0.22 μm微孔滤膜过滤即得供试品溶液。

#### 1.2.2 定性分析的液相色谱条件

色谱柱:Waters ACQUITY UPLC BEH C18色谱柱(100 mm×2.1 mm, 1.7 μm);流动相A为0.1%(v/v)甲酸水溶液,流动相B为乙腈。梯度洗脱条件:0~2 min, 10%B~25%B; 2~7 min, 25%B~32%B; 7~10 min, 32%B~33%B; 10~12 min, 33%B~35%B; 12~22 min, 35%B~46%B; 22~27 min, 46%B~56%B; 27~28.5 min, 56%B~95%B; 28.5~30 min, 95%B; 30~30.5 min, 95%B~10%B; 30.5~33 min, 10%B。流速为0.5 mL/min;柱温为40 ℃;进样体积2 μL。

#### 1.2.3 飞行时间质谱条件

电喷雾电离(ESI)源,负离子模式,离子源温度为110 ℃,脱溶剂气体为氮气,流速为850 L/h,温度为450 ℃,毛细管电压为2.5 kV,锥孔电压为50 V,低能量扫描时能量为6 eV,高能量扫描时能量为35~65 eV,扫描范围为*m/z* 100~1500。精确质量数用亮氨酸-脑啡肽(leucine-enkephalin)溶液进行校正。

### 1.3 含量测定

#### 1.3.1 对照品溶液的配制

分别精密称取对照品粉末绞股蓝皂苷XLVI和LVI适量,用乙醇-水(50∶50, v/v)溶解,于10 mL容量瓶中定容,制得质量浓度分别为1.272 mg/mL和1.636 mg/mL的对照品储备溶液,4 ℃冰箱保存备用。

#### 1.3.2 供试品溶液的配制

精密称取样品粉末0.2 g,置于100 mL具塞磨口锥形瓶中,加入30 mL乙醇-水-氨水(50∶46∶4, v/v/v),密塞,超声提取30 min(功率600 W,频率40 kHz),静置24 h,称重比较前后重量差异,加入提取溶剂补足减失的量,取1 mL上清液经0.22 μm微孔滤膜过滤,待测。

#### 1.3.3 含量测定的液相色谱条件

色谱柱:Waters ACQUITY UPLC BEH C18色谱柱(100 mm×2.1 mm, 1.7 μm);流动相A为0.1%(v/v)甲酸水溶液,流动相B为乙腈。梯度洗脱条件:0~10.4 min, 30%B; 10.4~10.5 min, 30%B~95%B; 10.5~14.5 min, 95%B; 14.5~14.6 min, 95%B~30%B; 14.6~20 min, 30%B。流速为0.5 mL/min;柱温为40 ℃;进样体积2 μL; 电雾式检测器采集频率10 Hz,蒸发温度35 ℃,滤波1 s。

## 2 结果与讨论

### 2.1 主成分鉴定

为充分了解福建产绞股蓝的化学成分信息,利用UHPLC-CAD分析福建产绞股蓝,得到其CAD指纹图谱,如图2a所示,发现色谱峰1~4为福建产绞股蓝的主要成分。在相同指纹图谱液相色谱条件下,利用UHPLC-Q-TOF/MS对福建产绞股蓝进行化学成分鉴定,基峰图如图2b所示。通过化合物的保留时间、相对分子质量、化学式、质量数误差、高能量裂解碎片等信息,结合对照品及相关文献报道即可鉴定出各个色谱峰对应的可能的化合物,鉴定结果见表2。

**图 2:**
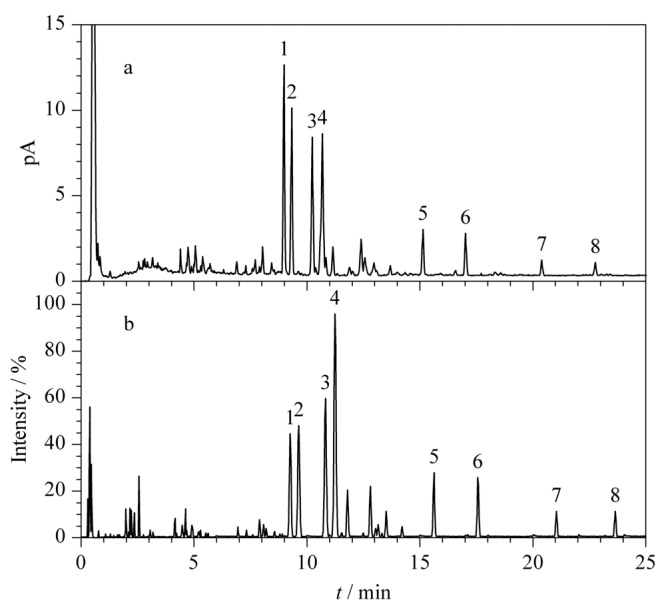
绞股蓝样品的(a) UHPLC-CAD分析色谱图和(b) UHPLC-Q-TOF/MS分析基峰图

**表 2:** 绞股蓝样品中主要的化学成分

No.	t_R_/ min	[M+HCOO]^-^ (m/z)	[M-H]^-^ (m/z)	Formula	Mass error (mDa)	Fragment ions (m/z)	Identification	Ref.
1	9.31	1139.5815	1093.5768	C_53_H_90_O_23_	-2.7	931.5248, 799.4843, 637.4322, 475.3796	gypenoside LVI	standard
2	9.68	-	1179.5765	C_56_H_92_O_26_	-3.4	1135.5875, 1093.5769, 931.5251, 799.4869,	malonylgypenoside	[11]
						637.4330, 475.3795	LVI	
3	10.86	1007.5393	961.5349	C_48_H_82_O_19_	-2.3	799.4833, 637.4313, 475.3790	gypenoside XLVI	standard
4-1	11.28	-	1047.5348	C_51_H_84_O_22_	-2.8	1003.5456, 961.5353, 799.4845, 637.4318,	malonylgypenoside	[11]
						475.3795	XLVI	
4-2	11.28	1123.5870	1077.5837	C_53_H_90_O_22_	0.8	945.5414, 783.4849, 459.3837	gypenoside IV	[26]
5	15.64	977.5294	931.5249	C_47_H_80_O_18_	-1.7	799.4980, 637.4367, 475.3831	gypenoside LVII	[27]
6	17.58	845.4882	799.4839	C_42_H_72_O_14_	-0.5	637.4319, 475.3790	gypenoside XLV	[28]
7	21.03	815.4780	769.4724	C_41_H_70_O_13_	-1.4	637.4319, 475.3820	gypenoside LXXVII	[29]
8	23.63	683.4365	637.4297	C_36_H_62_O_9_	-1.9	475.3859	gynosaponin TN1	[30]

以色谱峰3和4为例,具体鉴定过程如下。色谱峰3在负离子低能量模式下产生加合离子峰*m/z* 1007.5393 [M+HCOO]^-^和准分子离子峰*m/z* 961.5349 [M-H]^-^,推断其分子式为C_48_H_82_O_19_,同时,其可在负离子高能量模式下产生碎片离子*m/z* 799.4833、637.4313和475.3790,表明结构中含有3分子六碳糖。经与对照品保留时间、相对分子质量等信息对比确定其为绞股蓝皂苷XLVI。在负离子模式下色谱峰4产生了两组碎片离子(见图3),分别为*m/z* 1047.5348 [M_4-1_-H]^-^,以及*m/z* 1077.5837 [M_4-2_-H]^-^和1123.5870 [M_4-2_+HCOO]^-^的准分子离子峰,推测色谱峰4含有两个化合物,即化合物4-1和化合物4-2,根据其准分子离子峰,分别推断化合物4-1的分子式为C_51_H_84_O_22_ (*m/z* 1047.5348 [M_4-1_-H]^-^),化合物4-2的分子式为C_53_H_90_O_22_ (*m/z* 1077.5837 [M_4-2_-H]^-^)。通过提取准分子离子峰,化合物4-1在负离子高能量模式下产生碎片*m/z* 1003.5456、961.5353、799.4845、637.4318、475.3795,表明其在高能量时连续脱去中性特征碎片*m/z* 44(CO_2_)、*m/z* 42(C_2_H_2_O)和3分子六碳糖。除脱去*m/z* 44(CO_2_)、*m/z* 42(C_2_H_2_O)外,其他碎片与对照品绞股蓝皂苷XLVI一致,结合文献^[11]^报道推断化合物4-1为丙二酰基-绞股蓝皂苷XLVI。化合物4-2在负离子高能量模式下产生碎片*m/z* 945.5414、783.4849、459.3837,表明其在高能量时连续脱去1分子五碳糖和3分子六碳糖,结合文献报道^[26]^推断化合物4-2为绞股蓝皂苷IV。因此色谱峰4中包含了在此色谱条件下未能实现分离的化合物丙二酰基-绞股蓝皂苷XLVI和绞股蓝皂苷IV。

**图 3:**
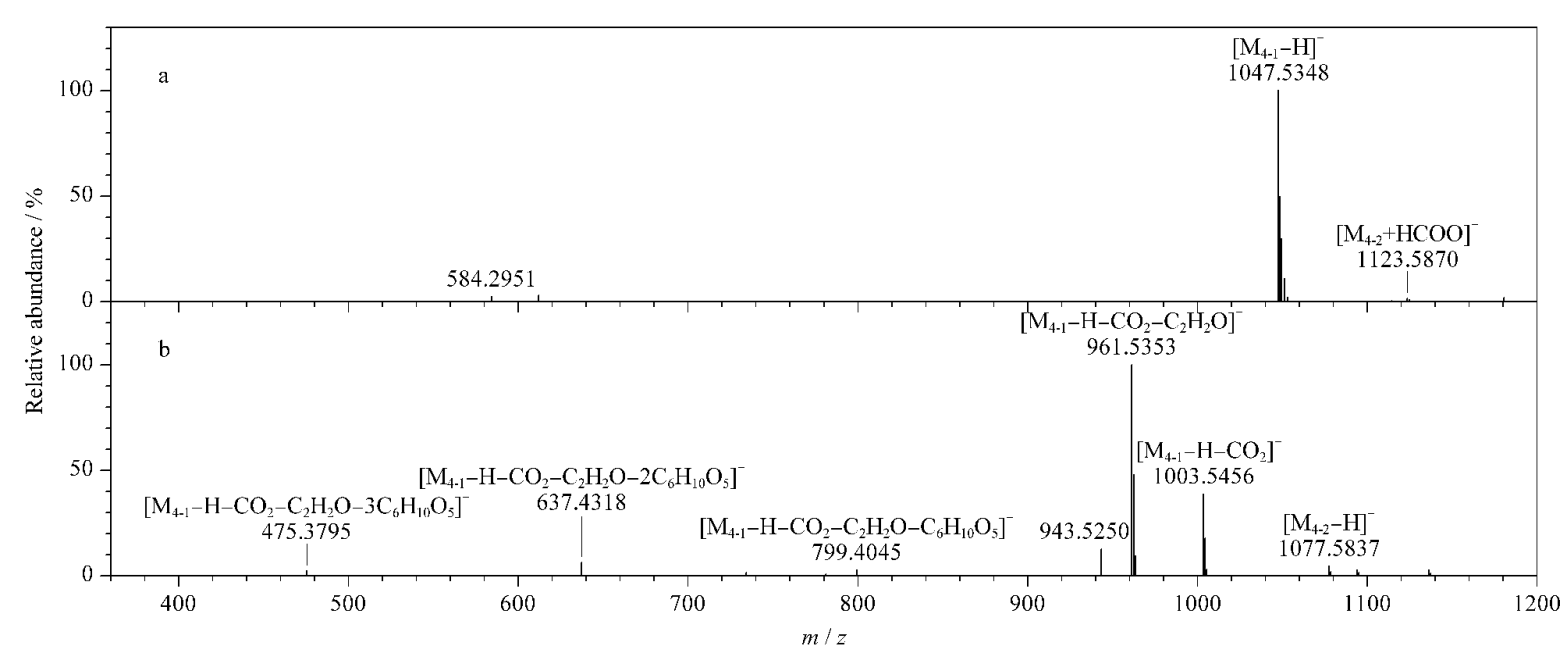
色谱峰4在负离子(a)低能量和(b)高能量模式下的质谱图

### 2.2 碱水解条件优化

丙二酰基-绞股蓝皂苷XLVI和LVI在福建产绞股蓝中含量较大,但其不稳定容易脱去丙二酰基,难以得到对照品,故参考人参含量测定文献中对丙二酰基酸性皂苷的处理方法^[31]^,适当进行条件优化后,对绞股蓝样品进行碱水解预处理,再进行含量测定。

分别考察了碱水解中氨水的体积分数(1%、2%和4%)和反应时间(12、24和36 h)对绞股蓝皂苷XLVI、绞股蓝皂苷LVI、丙二酰基-绞股蓝皂苷XLVI和丙二酰基-绞股蓝皂苷LVI含量的影响。当氨水体积分数为1%和2%时,丙二酰基-绞股蓝皂苷XLVI和丙二酰基-绞股蓝皂苷LVI在36 h内均未能完全转化,耗时过长;当氨水体积分数为4%时,24 h内丙二酰基-绞股蓝皂苷XLVI和LVI即可完全转化,且36 h与24 h无明显变化。综合考虑样品溶液的pH值以及反应时长,确定碱水解条件:氨水体积分数为4%,反应24 h。判断碱水解是否完全的过程如图4所示,分别为该条件下碱水解前、后的UHPLC-CAD色谱图,色谱峰2已完全转化,而峰面积相当的色谱峰4没有完全转化,由2.1节已知色谱峰4中包含了2个化合物,其中化合物4-2(绞股蓝皂苷IV)结构中不含有丙二酰基,碱水解对其无影响,结合质谱测定碱水解后该色谱峰*m/z* 1077.5837 [M-H]^-^为化合物4-2,说明含丙二酰基的化合物4-1已全部转化为去丙二酰基的绞股蓝皂苷XLVI,即该条件下碱水解已完全。

**图 4:**
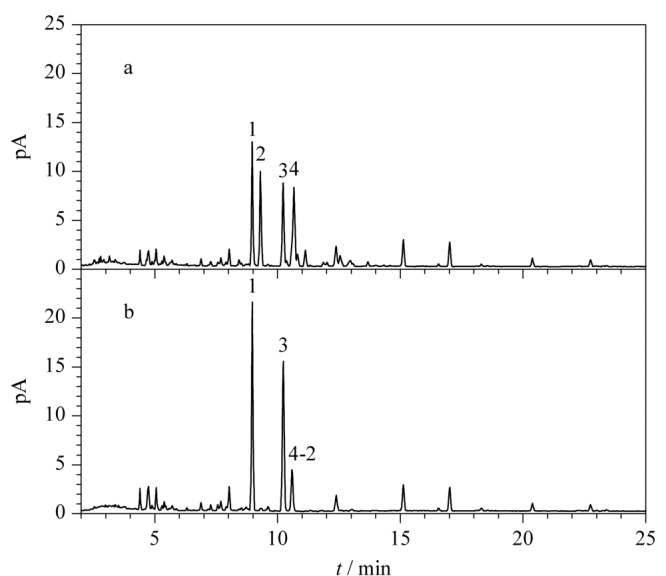
绞股蓝样品S10在含4%氨水提取溶剂中反应24 h(a)前、(b)后的色谱图

此外,取不同年份、不同来源和不同规格的福建产绞股蓝样品S1、S3、S6和S16在上述条件下进行碱水解,反应后分别取样品适量,在1.2.2节液相色谱条件下进行UHPLC-CAD分析。如图5所示,不同批次福建产绞股蓝样品的一致性较好,该碱水解条件下不同含量的丙二酰基-绞股蓝皂苷XLVI和LVI均能完全转化为相应的绞股蓝皂苷XLVI和LVI,且转化后绞股蓝皂苷XLVI和LVI的总含量远高于其他皂苷成分,进一步验证了优化后的碱水解条件的通用性以及质量评价指标成分选择的合理性。

**图 5:**
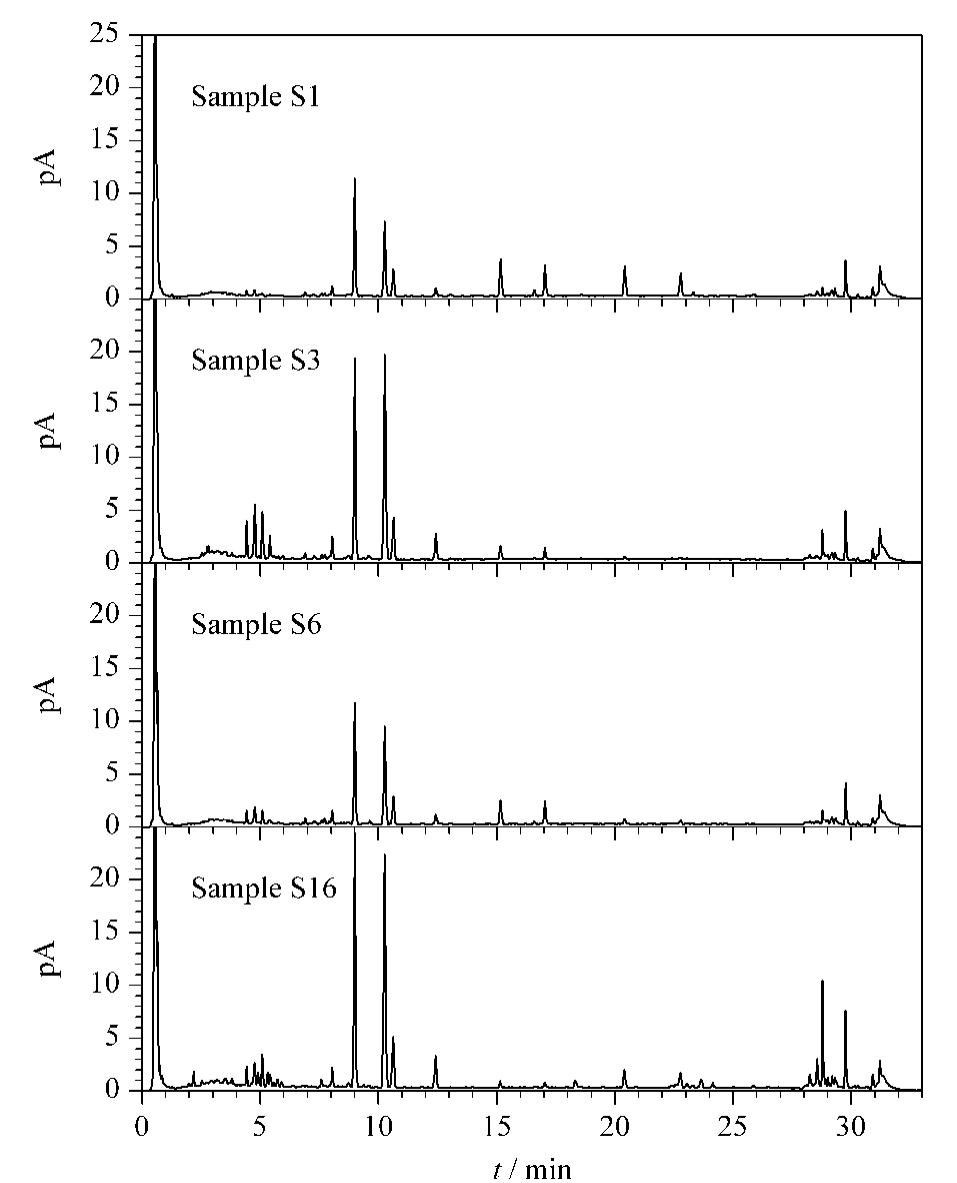
碱水解后不同批次绞股蓝样品的UHPLC-CAD色谱图

### 2.3 提取方法优化

在样品提取的过程中,分别考察了不同提取溶剂、提取料液比以及提取时间对绞股蓝皂苷XLVI和LVI提取率的影响。同时,结合2.2节碱水解条件优化结果,在提取方法优化过程中均在提取溶剂中加入体积分数为4%的氨水。当料液比为1∶150(g∶mL)、超声提取时间为30 min时,在不同提取溶剂(30%、50%、70%甲醇水溶液和30%、50%、70%乙醇水溶液)下,总提取液中绞股蓝皂苷XLVI和LVI的峰面积之和分别为13378、22305、24015、17670、23959和21043,其中,70%甲醇水溶液与50%乙醇水溶液对两种成分的提取率相当,但由于乙醇更加绿色环保,故选用50%乙醇水溶液(含4%氨水)作为提取溶剂。

当提取溶剂为50%乙醇水溶液(含4%氨水),提取时间为30 min时,在不同料液比(1∶50、1∶100、1∶150和1∶200, g∶mL)下,总提取液中绞股蓝皂苷XLVI和LVI的峰面积之和分别是18152、20721、23959和23974, 1∶150(g∶mL)与1∶200(g∶mL)料液比下,两种成分的提取率相当,综合考虑样品浓度等因素,确定料液比为1∶150(g∶mL)。

此外,当提取溶剂为50%乙醇水溶液(含4%氨水),提取料液比为1∶150(g∶mL),超声提取时间分别为20、30、40和50 min,总提取液中绞股蓝皂苷XLVI和LVI的峰面积之和分别是22888、23959、23917和23983,当提取时间为30 min时,两种化合物已被充分提取,再随着提取时间的增加,峰面积无明显差异,为缩短分析操作时间,提高分析效率,故选择提取时间为30 min。

最终提取条件确定为:乙醇-水-氨水(50∶46∶4, v/v/v)作为提取溶剂,料液比1∶150(g∶mL),超声提取30 min。

### 2.4 液相色谱条件的优化

考察了不同的色谱柱以及不同的流动相组成对供试品或对照品峰形和分离效果的影响。由于提取溶液中加入了氨水,供试品溶液具有一定的碱性(pH=8.8),故选择的色谱柱须耐受一定的碱性,选择Waters ACQUITY UPLC BEH C18色谱柱(100 mm×2.1 mm, 1.7 μm; pH耐受范围:1~12)。

关于流动相的组成,考察了不同的流动相系统(水-甲醇和水-乙腈)对化学成分分离效果的影响,结果显示在水-乙腈系统下,各待测成分分离效果较好,响应较高,同时,在实验中发现加入适量的甲酸有助于峰形的改善,因此最终确定流动相A为0.1%(v/v)甲酸水溶液,流动相B为乙腈。如图6所示,该液相色谱条件下供试品中绞股蓝皂苷XLVI和LVI保留适中,分离度良好。

**图 6:**
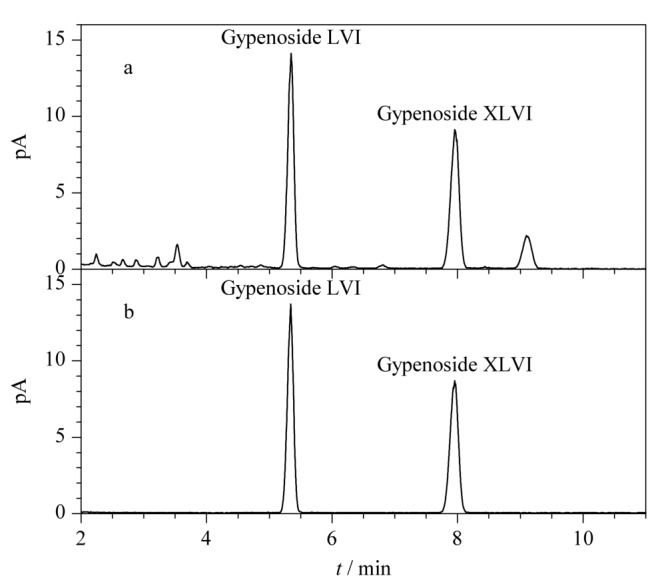
(a)绞股蓝样品和(b)混合对照品的UHPLC-CAD色谱图

### 2.5 方法学考察

#### 2.5.1 线性关系、检出限和定量限

精密吸取1.3.2节配制的对照品储备液适量,并将其均匀混合,用50%乙醇逐级稀释,制得绞股蓝皂苷XLVI质量浓度分别为318.00、159.00、79.50、39.75、19.88、9.94 μg/mL,绞股蓝皂苷LVI质量浓度分别为409.00、204.50、102.25、51.13、25.56、12.78 μg/mL的系列混合对照品溶液;精密吸取上述系列混合对照品溶液各2 μL,分别按1.3.3节的液相色谱条件进样测定。CAD是基于雾化-气溶胶的HPLC检测器,其响应值与被测物质的量呈指数关系,一般需经对数转化或用二次函数计算^[32]^。本文以待测组分质量浓度(μg/mL)的对数值(*X*)为横坐标,峰面积的对数值(*Y*)为纵坐标建立标准曲线。结果如表3所示,绞股蓝皂苷XLVI在9.94~318.94 μg/mL、绞股蓝皂苷LVI在12.78~409.00 μg/mL范围内,*X*与*Y*线性关系良好,相关系数(*r*)分别为0.9993和0.9995。

**表 3:** 绞股蓝皂苷XLVI与LVI的线性方程、线性范围、相关系数、检出限和定量限

Compound	Linear equation	Linear range (μg/mL)	r	LOD/(μg/mL)	LOQ/(μg/mL)
XLVI	Y=0.8334X-1.4707	9.94-318.00	0.9993	1.58	6.36
LVI	Y=0.7993X-1.4304	12.78-409.00	0.9995	2.05	8.18

*Y*: logarithm of peak area; *X*: logarithm of mass concentration.

分别对2种对照品储备液逐级稀释,进样检测,分别以信噪比(*S/N*)等于3和10为标准,确定2种化合物的检出限(LOD)和定量限(LOQ),其中,绞股蓝皂苷XLVI的检出限为1.58 μg/mL,定量限为6.36 μg/mL;绞股蓝皂苷LVI的检出限为2.05 μg/mL,定量限为8.18 μg/mL。

#### 2.5.2 精密度试验

取混合对照品溶液,按1.3.3节的液相色谱条件连续进样6次,记录峰面积。绞股蓝皂苷XLVI和LVI峰面积的RSD值分别为0.88%和0.44%(*n*=6),表明仪器精密度良好。

#### 2.5.3 稳定性试验

取1.3.2节方法制得的同一供试品溶液适量(编号:S10),分别在室温下放置0、2、4、6、8、12、24 h,按1.3.3节的液相色谱条件进样检测,记录峰面积,计算绞股蓝皂苷XLVI和LVI的含量。二者的RSD值分别为0.98%和0.90%(*n*=6),表明供试品溶液在24 h内稳定。

#### 2.5.4 重复性试验

精密称取6份同一批次的绞股蓝样品粉末(编号:S10)0.20 g,按1.3.2节方法制备供试品溶液,按1.3.3节的液相色谱条件连续进样,记录峰面积,计算绞股蓝皂苷XLVI和LVI的含量。二者RSD值分别为1.39%和1.55%(*n*=6),表明该方法的重复性良好。

#### 2.5.5 加标回收率试验

精密称取6份同一批次的绞股蓝粉末(编号:S10)0.10 g,精密加入相当量的对照品溶液,按1.3.2节方法制备供试品溶液,按1.3.3节液相色谱条件,连续进样检测,记录峰面积,计算绞股蓝皂苷XLVI和LVI的含量。绞股蓝皂苷XLVI与LVI的加标回收率分别在100.2%~107.2%与97.9%~104.2%范围内,RSD值分别为2.4%与2.6%,表明该方法准确性良好。

### 2.6 绞股蓝样品含量测定

对包括种植、野生和商品茶在内的16批福建产绞股蓝样品进行含量测定,分别精密称取样品粉末0.2 g,按1.3.2节方法制备供试品溶液,按1.3.3节液相色谱条件进样检测,分别测定各批次绞股蓝样品中绞股蓝皂苷XLVI和LVI的含量,结果见表4。绞股蓝中绞股蓝皂苷XLVI的含量在0.57~2.57%之间,绞股蓝皂苷LVI的含量在0.66~2.99%之间。其中,野生绞股蓝样品(编号:S1、S2、S6和S10)中指标成分含量略低于种植样品;商品茶样品(编号:S14、S15和S16)的指标成分含量高于其他样品。

**表 4:** 不同批次绞股蓝样品中绞股蓝皂苷XLVI和 LVI的含量(*n*=3)

Sample	XLVI			LVI	
	Content/%	RSD/%		Content/%	RSD/%
S1	0.57	3.23		0.84	2.56
S2	1.09	1.62		0.93	0.64
S3	1.88	3.55		1.61	2.37
S4	1.44	0.88		2.05	1.64
S5	1.52	0.85		1.98	0.07
S6	0.81	2.41		0.88	3.63
S7	1.17	0.66		0.96	0.76
S8	2.01	2.61		1.81	0.78
S9	1.68	1.85		2.27	1.46
S10	1.16	0.20		1.49	1.15
S11	1.17	1.95		0.98	1.68
S12	1.01	1.07		0.84	1.32
S13	1.15	1.16		0.66	0.79
S14	2.57	2.59		2.99	2.45
S15	2.16	1.83		2.05	1.70
S16	2.12	0.82		2.10	1.43

## 3 结论

本研究利用UHPLC-Q-TOF/MS结合UHPLC-CAD鉴定了福建产绞股蓝的主要成分,选择2个含量较高的共有成分绞股蓝皂苷XLVI和LVI作为指标成分,利用UHPLC-CAD建立了福建产绞股蓝中绞股蓝皂苷XLVI和LVI含量的测定方法。该方法灵敏度高,重复性好,可用于福建产绞股蓝的质量评价和质量控制,为福建产绞股蓝质量标准的建立和完善提供了方法参考。
